# Diverse patient trajectories during cytotoxic chemotherapy: Capturing longitudinal patient‐reported outcomes

**DOI:** 10.1002/cam4.4124

**Published:** 2021-07-13

**Authors:** Amee D. Azad, Melih Yilmaz, Selen Bozkurt, James D. Brooks, Douglas W. Blayney, Tina Hernandez‐Boussard

**Affiliations:** ^1^ Department of Medicine Stanford University School of Medicine Stanford California USA; ^2^ Department of Medicine (Biomedical Informatics Stanford University School of Medicine Stanford California USA; ^3^ Department of Urology Stanford University School of Medicine Stanford California USA; ^4^ Department of Medicine Division of Medical Oncology Stanford University School of Medicine Stanford California USA; ^5^ Department of Biomedical Data Science Stanford University School of Medicine Stanford California USA

**Keywords:** chemotherapy, global mental health, global physical health, patient trajectories, patient‐reported outcomes, PROMIS

## Abstract

**Background:**

High‐value cancer care balances effective treatment with preservation of quality of life. Chemotherapy is known to affect patients’ physical and psychological well‐being negatively. Patient‐reported outcomes (PROs) provide a means to monitor declines in a patients’ well‐being during treatment.

**Methods:**

We identified 741 oncology patients undergoing chemotherapy in our electronic health record (EHR) system who completed Patient‐Reported Outcomes Measurement Information System (PROMIS) surveys during treatment at a comprehensive cancer center, 2013–2018. PROMIS surveys were collected before, during, and after chemotherapy treatment. Linear mixed‐effects models were performed to identify predictors of physical and mental health scores over time. A k‐mean cluster analysis was used to group patient PROMIS score trajectories.

**Results:**

Mean global physical health (GPH) scores were 48.7 (SD 9.3), 47.7 (8.8), and 48.6 (8.9) and global mental health (GMH) scores were 50.4 (8.6), 49.5 (8.8), and 50.6 (9.1) before, during, and after chemotherapy, respectively. Asian race, Hispanic ethnicity, public insurance, anxiety/depression, stage III cancer, and palliative care were predictors of GPH and GMH decline. The treatment time period was also a predictor of both GPH and GMH decline relative to pre‐treatment. Trajectory clustering identified four distinct PRO clusters associated with chemotherapy treatment.

**Conclusions:**

Patient‐reported outcomes are increasingly used to help monitor cancer treatment and are now a part of care reimbursement. This study leveraged routinely collected PROMIS surveys linked to EHRs to identify novel patient trajectories of physical and mental well‐being in oncology patients undergoing chemotherapy and potential predictors. Supportive care interventions in high‐risk populations identified by our study may optimize resource deployment.

**Novelty and impact:**

This study leveraged routinely collected patient‐reported outcome (PROMIS) surveys linked to electronic health records to characterize oncology patients’ quality of life during chemotherapy. Important clinical and demographic predictors of declines in quality of life were identified and four novel trajectories to guide personalized interventions and support. This work highlights the utility of monitoring patient‐reported outcomes not only before and after, but during chemotherapy to help advert adverse patient outcomes and improve treatment adherence.

## Graphical abstract

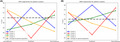

## 1 INTRODUCTION

Cancer and its treatment take a heavy physical and mental toll on patients. Chemotherapy induces adverse side effects that can significantly impact patients’ physical and psychological well‐being during and after treatment. One study of breast cancer survivors demonstrated a higher prevalence of post‐traumatic stress disorder and ongoing physical sequelae 20 years after receiving chemotherapy.^1^ Another study showed that cancer survivors without prior chemotherapy had a higher quality of life than those who had received systemic adjuvant therapy.^2^ While the primary goal of oncology is to eliminate cancer, understanding the patients’ lived experience throughout their treatment journey, such as daily functioning and mental health, is also a priority in a patient‐centered healthcare delivery system.^3^, ^4^ Furthermore, incorporation of patient‐reported outcomes (PROs) into clinical care pathways can improve the patient experience and patient outcomes,^5^, ^6^, ^7^, ^8^, ^9^ including survival, and has been increasingly recognized as an important metric in clinical trials.^6^, ^10^, ^11^, ^12^, ^13^, ^14^, ^15^ PRO collection for symptom monitoring during cancer treatment is also a new requirement with the Oncology Care First alternative payment model from the Centers for Medicare & Medicaid Services (CMS) that rolled out at the end of 2020.^16^ Consequently, PROs will have greater utility in research and practice, forming a central tenet to clinical outcome evaluation, and are positioned to provide vital insight into patients’ experience.

The Patient‐Reported Outcomes Measurement Information System (PROMIS) survey, initiated by the National Institutes of Health and intended to be used in any disease or clinical population, provides a systematic approach for measuring patients’ physical and mental well‐being across any type of medical treatment.^17^, ^18^ Prior studies have offered important considerations in PRO implementation,^4^, ^12^ particularly related to the generalizability of generic measures to specific patient populations, such as cancer patients with metastatic disease.^19^, ^20^ Other studies have focused on the evaluation of PROs at a specific time point during treatment or through cross‐sectional study designs.^21^, ^22^, ^23^ These snapshots of symptoms have demonstrated great variability in both physical and mental health average scores across patient demographics, cancer types, and treatment pathways.^24^, ^25^ Some studies that have collected PROMIS surveys longitudinally aimed to predict symptom clusters or validate PROMIS scores, but were limited by small sample sizes.^26^, ^27^, ^28^, ^29^, ^30^, ^31^, ^32^, ^33^ However, a paucity of literature exists on risk factors associated with quality of life *during* chemotherapy administration, which is crucial to identify and target for intervention. Importantly, there is a gap in evidence on patients’ physical and mental health trajectory during treatment that requires substantial longitudinal data at multiple time points.^34^, ^35^, ^36^, ^37^ Furthermore, linking this quality‐of‐life data to a patient’s electronic health record is essential to improve the understanding of declines in physical and mental health. This study provides the groundwork to improve the understanding of what PRO measurement, now a requirement of CMS’ Oncology Care First model, can add to our understanding of longitudinal cancer care. We also aim to support future studies that aggregate these data for quality assessment, comparative‐effectiveness research, and randomized controlled trials.

The goals of this study were to leverage the PROMIS global survey to (1) identify patients who are vulnerable to declines in PROMIS score following chemotherapy based on pre‐treatment PROMIS scores and baseline characteristics; and (2) characterize patients’ physical and mental health trajectories during chemotherapy treatment. The aim of this study is to guide quality improvement efforts, resource allocation, future interventional studies, and to improve cancer patients’ overall experience.

## 2 MATERIALS AND METHODS

### 2.1 Data source

This retrospective observational study used EHR (electronic health records) which were first implemented in 2008. The cohort includes patients from an academic medical center, a community hospital, and a healthcare alliance that includes primary and specialty care clinics. The EHR chemotherapy ordering module was implemented in 2009, and integration of PROMIS surveys into the outpatient clinic workflow occurred in 2013, as previously described.^24^ The institute’s Institutional Review Board approved the study.

### 2.2 Participants

**FIGURE 1:**
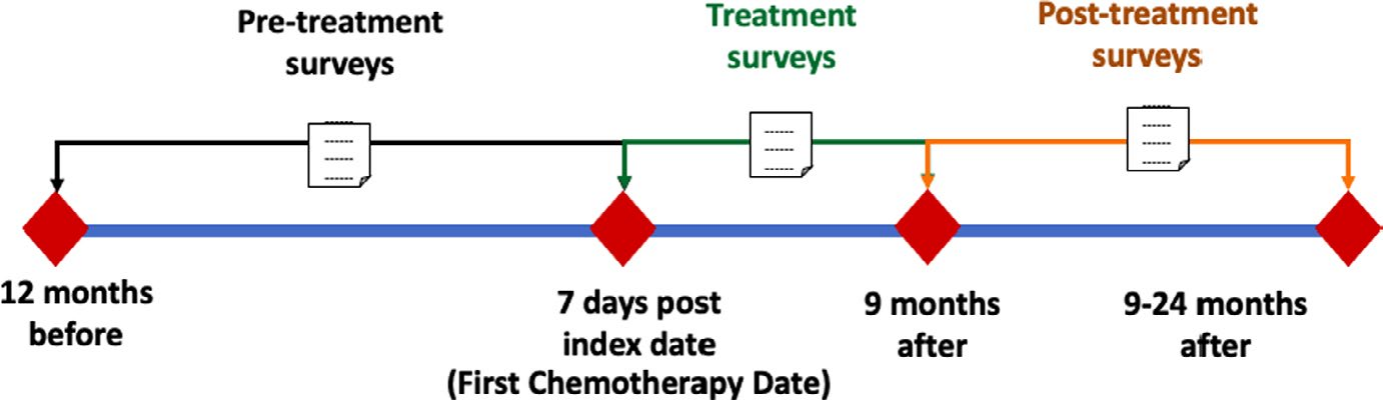
Patient‐Reported Outcomes Measurement Information System (PROMIS) survey collection intervals for patients undergoing chemotherapy treatment

This study included cancer patients from 2013 to 2018. A patient’s index date was set as the date of their first chemotherapy session as defined by the International Classification of Disease, 9th and 10th Edition (ICD 9/10) and Current Procedural Terminology (CPT) codes (Figure S1). Patients were included if they completed one PROMIS survey in each of the three‐time intervals (therefore, requiring at least three total surveys) before, during, and after chemotherapy (Figure 1). Pre‐treatment surveys were those collected from 12 months preceding to 7 days following the index date, treatment surveys were collected between the 7 days following the index date and 9 months afterward, and post‐treatment surveys were collected 9–24 months after the index date. The additional 7 days following the index date for pre‐treatment surveys were included to account for a known lag in recording patients’ therapy plans in the EHR. If there were multiple surveys during a time period, we chose the survey closest to the index date since PROMIS surveys were collected at each outpatient visit.

### 2.3 Study variables

Study variables included time of survey (pre‐treatment, treatment, or post‐treatment), sex, age at diagnosis, race, ethnicity, insurance status, anxiety/depression diagnosis, stage of cancer at diagnosis, treatment goal (palliative or curative), and cancer type. Patients with anxiety/depression were identified by ICD 9/10 codes for anxiety/depression at any point during the study period (Table S2).^38^ Treatment goal is a designation in the EHR by treating oncologist, a required field in the EHR—either curative or palliative—prior to chemotherapy initiation.

### 2.4 Outcome measures

The primary outcomes were PROMIS survey global physical health and global mental health scores. PROMIS responses were mapped on a one to five scale with 1 being poor and five being excellent, except for pain which was rated on a 1–10 scale (higher scores indicating worse pain). Global physical health and global mental health scores were each converted to standardized T‐scores, according to PROMIS guidelines.^18^ The T‐score rescales the raw sum score into a standardized score with a mean of 50 and a standard deviation (SD) of 10. The T‐score has previously been calibrated to a mean of 50 for global physical health and global mental health for a random sample of healthy people from the U.S. population, with higher scores indicating better health.^17^

### 2.5 Statistical analysis

#### 2.5.1 Global physical health and global mental health scores

Patient demographics and clinical characteristics before, during, and after treatment PROMIS scores were compared using repeated‐measures ANOVA. Linear mixed‐effects models were used to identify factors associated with global physical and mental health scores with a random slope for each patient to account for within‐subject variation in PROMIS score over time.^39^ Patients were excluded from the multivariable modeling for missing demographic data (sex, age at diagnosis, race/ethnicity, insurance status) or clinical data (prior to the chemotherapy diagnosis of anxiety/depression, stage of cancer at diagnosis), treatment goal (curative or palliative chemotherapy, and cancer type). ‘Other’ cancer types included neurologic cancers and sarcomas and were combined due to low sample sizes. A purposeful variable selection method was employed for multivariate analysis (variables that reached significance in the univariable models were included for the multivariable models)^40^ and a complete case analysis was conducted for each model. *p*‐values <0.05 were considered significant. All analyses were conducted using R version 3.6.1 (R Foundation for Statistical Computing). The R package ‘nlme’ was used for variable selection in the multivariable analysis.

#### 2.5.2 Clustering and prediction of patient trajectories

K‐means cluster analysis was used to classify patient PROMIS score trajectories across their treatment course. Two features were used in the cluster analysis: the difference between pre‐treatment and treatment survey scores and the difference between treatment and post‐treatment survey scores for global physical and mental health. The total number of clusters was determined by the elbow method.^41^ K‐mean clustering was implemented in Python using the scikit‐learn v0.21.3.

#### 2.5.3 Sensitivity analysis

As a sensitivity analysis, we compared this cohort to chemotherapy patients who did not complete at least three PROMIS surveys during the specified time periods. Patient demographics and clinical characteristics were compared using chi‐square and Student *t*‐test analyses.

## 3 RESULTS

### 3.1 Demographics and survey characteristics

Of the 741 patients in this study, 438 (59.1%) were female, with an average age at diagnosis of 59.1 years (SD 13.1). Four hundred and seventy‐seven patients (64.4%) were non‐Hispanic White, 141 (19.0%) were Asian, and 364 (49.1%) were privately insured. One hundred and forty‐four patients (19.4%) had a diagnosis of anxiety/depression prior to initiation of chemotherapy. Patients were most commonly diagnosed in stage I (181 [24.4%]) and were undergoing curative therapy (543 [73.3%]). Breast cancer (29.0%) and lymphoma/leukemia (18.1%) were among the most common cancer types (Table 1). The average time that surveys were collected prior to treatment was 32.7 days (SD 55.1 days), treatment surveys were collected 92.9 days (SD 57.3 days) after initiation of treatment, and post‐treatment surveys were collected at 366.2 days (SD 92.3 days) after initiation of treatment.

**TABLE 1:** Patient demographics and clinical characteristics before, during, and after chemotherapy, stratified by physical and mental health scores

	*N* (%), mean (SD)	Physical health scores				Mental health scores			
		Pre‐treatment	Treatment	Post‐treatment		Pre‐treatment	Treatment	Post‐treatment	
		Mean (SD)	Mean (SD)	Mean (SD)	*p*‐value	Mean (SD)	Mean (SD)	Mean (SD)	*p*‐value
All patients	741 (100.0)	48.7 (9.3)	47.7 (8.8)	48.6 (8.9)	0.983	50.4 (8.6)	49.5 (8.8)	50.6 (9.1)	0.618
Age	59.1 (13.1)								
Sex					0.381				0.082
Male	303 (40.9)	49.0 (9.1)	47.8 (8.8)	48.9 (9.2)		50.6 (8.6)	49.7 (8.9)	51.3 (9.5)	
Female	438 (59.1)	48.6 (9.5)	47.7 (8.7)	48.4 (8.7)		50.2 (8.6)	49.3 (8.7)	50.2 (8.7)	
Race/ethnicity					0.079				0.866
Non‐hispanic white	477 (64.4)	48.4 (8.6)	48.4 (8.6)	49.3 (8.7)		51.7 (8.6)	50.6 (8.8)	51.9 (9.2)	
Hispanic	61 (8.2)	43.7 (9.5)	44.4 (7.6)	47.4 (8.7)		46.6 (8.2)	47.1 (8.4)	48.7 (8.0)	
Asian	141 (19.0)	46.6 (9.6)	47.3 (9.4)	47.6 (8.3)		48.3 (7.6)	47.9 (8.6)	48.6 (8.3)	
Other	55 (7.4)	48.2 (9.8)	46.3 (8.2)	45.9 (11.0)		48.1 (8.8)	47.3 (8.1)	47.3 (9.3)	
Insurance					0.236				0.684
Private	364 (49.5)	49.6 (9.3)	48.4 (8.4)	50.2 (8.3)		50.7 (8.5)	49.5 (8.7)	50.7 (8.8)	
Medicare	351 (47.8)	48.1 (9.4)	47.3 (8.9)	46.7 (9.5)		50.3 (8.5)	49.7 (8.6)	50.2 (9.8)	
Medicaid	20 (2.7)	42.8 (7.5)	44.1 (8.9)	45.5 (11.3)		43.7 (9.8)	44.2 (9.5)	45.6 (11.1)	
Anxiety/depression diagnosis					0.971				0.841
Diagnosis	144 (19.4)	45.8 (9.5)	45.0 (8.4)	45.7 (9.1)		46.2 (8.8)	45.6 (8.0)	46.3 (9.2)	
No diagnosis	597 (80.6)	49.5 (9.2)	48.4 (8.7)	49.3 (8.7)		51.4 (8.2)	50.4 (8.7)	51.7 (8.7)	
Stage at diagnosis					0.750				0.598
I	181 (30.5)	51.1 (8.6)	48.6 (8.7)	50.2 (7.9)		50.6 (9.5)	49.3 (8.7)	51.2 (9.0)	
II	168 (28.3)	48.6 (9.3)	48.1 (8.6)	48.8 (8.4)		50.9 (7.9)	49.9 (8.6)	50.0 (9.1)	
III	117 (19.7)	49.3 (9.1)	48.9 (8.7)	49.8 (9.5)		50.6 (8.7)	49.7 (7.9)	50.6 (9.5)	
IV	128 (21.5)	46.5 (8.9)	46.8 (8.4)	47.4 (9.3)		50.4 (9.3)	49.6 (9.1)	49.4 (9.8)	
Treatment goal					0.351				0.500
Curative	543 (73.3)	49.6 (9.1)	48.4 (8.7)	49.6 (8.6)		50.8 (8.5)	50.1 (8.6)	51.4 (9.0)	
Palliative	198 (26.7)	46.3 (9.5)	45.9 (8.6)	45.6 (9.0)		49.1 (8.7)	47.8 (8.9)	48.5 (8.9)	
Cancer type					0.476				0.758
Breast	215 (29.0)	50.6 (8.8)	48.4 (8.7)	48.9 (7.6)		51.3 (8.0)	49.6 (8.6)	50.6 (8.1)	
Lymphoma/leukemia	134 (18.1)	48.2 (9.5)	48.4 (8.6)	48.0 (8.6)		50.6 (8.4)	50.8 (8.2)	51.4 (9.3)	
Gastrointestinal	76 (10.3)	48.3 (9.6)	48.8 (8.3)	49.6 (10.0)		49.7 (7.3)	50.4 (8.2)	50.1 (8.5)	
Head and neck	75 (10.1)	49.9 (8.1)	46.4 (8.2)	49.2 (9.7)		52.8 (9.1)	49.7 (9.0)	51.6 (10.1)	
Genitourinary	63 (8.5)	49.6 (9.3)	48.3 (8.9)	48.7 (8.3)		50.3 (8.1)	50.3 (9.1)	51.1 (8.6)	
Lung	54 (7.3)	45.5 (9.7)	46.6 (9.2)	45.7 (9.9)		47.1 (9.5)	47.2 (8.8)	48.9 (10.6)	
Gynecologic	41 (5.5)	47.7 (9.5)	47.3 (8.7)	49.7 (8.7)		49.6 (10.1)	48.4 (9.1)	51.2 (8.3)	
Skin	31 (4.2)	48.3 (10.2)	47.8 (10.4)	49.2 (10.5)		49.7 (10.3)	49.4 (9.3)	49.6 (11.0)	
Hepatobiliary/pancreatic	30 (4.0)	44.6 (10.8)	45.5 (9.2)	47.6 (9.3)		48.6 (7.8)	47.4 (8.4)	48.4 (7.4)	
Other**	22 (2.9)	44.7 (9.4)	44.3 (8.8)	47.7 (9.9)		48.1 (8.5)	46.3 (9.5)	49.7 (9.7)	

** ‘Other’ cancer = neurologic and sarcoma patients.

In one sensitivity analysis, 6364 patients did not complete PROMIS surveys. Patients who did not complete a PROMIS survey were significantly different relative to our study cohort with regards to age, sex, race/ethnicity, insurance type, stage at diagnosis, and cancer type (Table S3).

### 3.2 Global physical and mental health score trajectories

Table 1 also shows the global health and mental health scores over time. The mean global physical health score for all patients was 48.7 (SD 9.3) before treatment, 47.7 (SD 8.8) in the treatment phase, and 48.6 (SD 8.9) in the post‐treatment phase (*p *= 0.983). The average global mental health score across all cancer types was 50.4 (SD 8.6) before treatment, 49.5 (SD 8.8) in the treatment phase, and 50.6 (SD 9.1) in the post‐treatment phase, and also did not differ significantly over time (*p *= 0.618) (Table 1). Physical and mental health score trajectories varied greatly by cancer type (Figure S2A,B).

We found that 40.5% of patients had a clinically significant decline (CSD) in physical health score, defined as a three‐point change in score,^42^ between pre‐treatment and treatment time periods. We also found that 32.1% declined between the post‐treatment and treatment time periods and 38.6% between the post‐treatment and pre‐treatment time periods for physical health scores. For mental health scores, we observed a CSD for 32.7% of patients between pre‐treatment and treatment, 25.5% between treatment and post‐treatment, and 31.3% between pre‐treatment and post‐treatment time periods.

### 3.3 Factors associated with global physical and mental health scores

Multivariable linear mixed‐effects modeling for global physical health showed significantly lower scores for those of Asian race (*β *= −2.30, *p *= 0.002) and Hispanic ethnicity (*β *= −3.10, *p *= 0.006) compared to non‐Hispanic White patients, as well as those with Medicaid and Medicare compared to patients who were privately insured (*β *= −3.89, *p *= 0.027 and *β *= −1.74, *p *= 0.005, respectively), a pre‐chemotherapy diagnosis of anxiety/depression compared those without (*β* = −4.09, *p *< 0.001), and stage III compared to stage I at diagnosis (*β* = −1.82, *p *= 0.039). Palliative treatment was also found to be associated with lower physical health scores compared to curative treatment (*β* = −2.93, *p *< 0.001) and the treatment time period was associated with significantly lower scores compared to the pre‐treatment time period (*β *= −1.12, *p *= 0.002), but no difference was observed at post‐treatment. There were no significant differences in the score for cancer type for physical health. Sex and age at diagnosis did not show a significant difference in the univariate analysis and was therefore not included in the multivariable model (Table 2).

**TABLE 2:** Linear mixed‐effects model results for physical health scores

	Coefficient	Standard error	*p*‐value
Time period			
Pre‐treatment	Reference		
Treatment	−1.12	0.36	0.002
Post‐treatment	−0.10	0.40	0.794
Race/ethnicity			
Non‐hispanic white	Reference		
Hispanic	−3.10	1.12	0.006
Asian	−2.30	0.74	0.002
Other	2.50	2.4	0.299
Insurance			
Private	Reference		
Medicaid	−3.89	1.76	0.027
Medicare	−1.74	0.62	0.005
Anxiety/depression diagnosis			
No diagnosis	Reference		
Diagnosis	−4.09	0.744	<0.001
Stage at diagnosis			
I	Reference		
II	−1.08	0.77	0.159
III	−1.82	0.88	0.039
IV	−1.41	0.94	0.134
Treatment goal			
Curative	Reference		
Palliative	−2.93	0.72	<0.001
Cancer type			
Breast	Reference		
Lymphoma/leukemia	1.02	1.19	0.394
Gastrointestinal	0.75	1.03	0.464
Head and neck	−0.38	1.12	0.734
Genitourinary	−0.07	1.09	0.947
Lung	−0.97	1.25	0.438
Gynecologic	0.20	1.27	0.872
Skin	0.83	1.40	0.555
Hepatobiliary/pancreatic	−1.19	1.49	0.425
Other	2.50	2.40	0.299

Global mental health linear mixed‐effects modeling showed significantly lower scores during the treatment time period compared to pre‐treatment (*β* = −0.77, *p *= 0.006), but similarly no difference post‐treatment. In addition, other factors associated with a decline in mental health score included Hispanic ethnicity (*β* = −3.29, *p *= 0.001), Asian race (*β* = −3.37, *p *< 0.001), those with Medicaid compared to privately insured patients (*β* = −4.16, *p *= 0.011), a pre‐chemotherapy diagnosis of anxiety/depression compared to patients without (*β* = −5.30, *p *< 0.001), and palliative care patients compared to curative care patients (*β* = −1.42, *p *= 0.022). Increasing age was associated with improved mental health scores for patients (*β* = 0.06, *p *= 0.040). Finally, an association with declining mental health scores was observed for patients with lung cancer (−2,86, *p *= 0.016) compared to breast cancer patients. Sensitivity analyses demonstrated that this effect among cancer types did not persist when the reference was genitourinary cancer. Sex and stage did not reach significance in the univariate analysis for global mental health (Table 3).

**TABLE 3:** Linear mixed‐effects model for mental health scores

	Coefficient	Standard error	*p*‐value
Time period			
Pre‐treatment	Reference		
Treatment	−0.77	0.28	0.006
Post‐treatment	0.38	0.30	0.212
Age	0.06	0.03	0.040
Race/ethnicity			
Non‐hispanic white	Reference		
Hispanic	−3.29	0.97	0.001
Asian	−3.37	0.70	<0.001
Other	−3.60	1.01	<0.001
Insurance			
Private	Reference		
Medicaid	−4.16	1.63	0.011
Medicare	−1.17	0.72	0.103
Anxiety/depression diagnosis			
No diagnosis	Reference		
Diagnosis	−5.30	0.67	<0.001
Treatment goal			
Curative	Reference		
Palliative	−1.42	0.62	0.022
Cancer type			
Breast	Reference		
Lymphoma/leukemia	−0.73	0.89	0.416
Gastrointestinal	−0.68	1.04	0.512
Head and neck	−0.12	1.09	0.916
Genitourinary	−0.78	1.18	0.510
Lung	−2.86	1.18	0.016
Gynecologic	−0.31	1.20	0.797
Skin	−0.91	1.41	0.521
Hepatobiliary/pancreatic	−2.64	1.44	0.068
Other	−2.78	1.65	0.093

### 3.4 Clustering of patient trajectories

**FIGURE 2:**
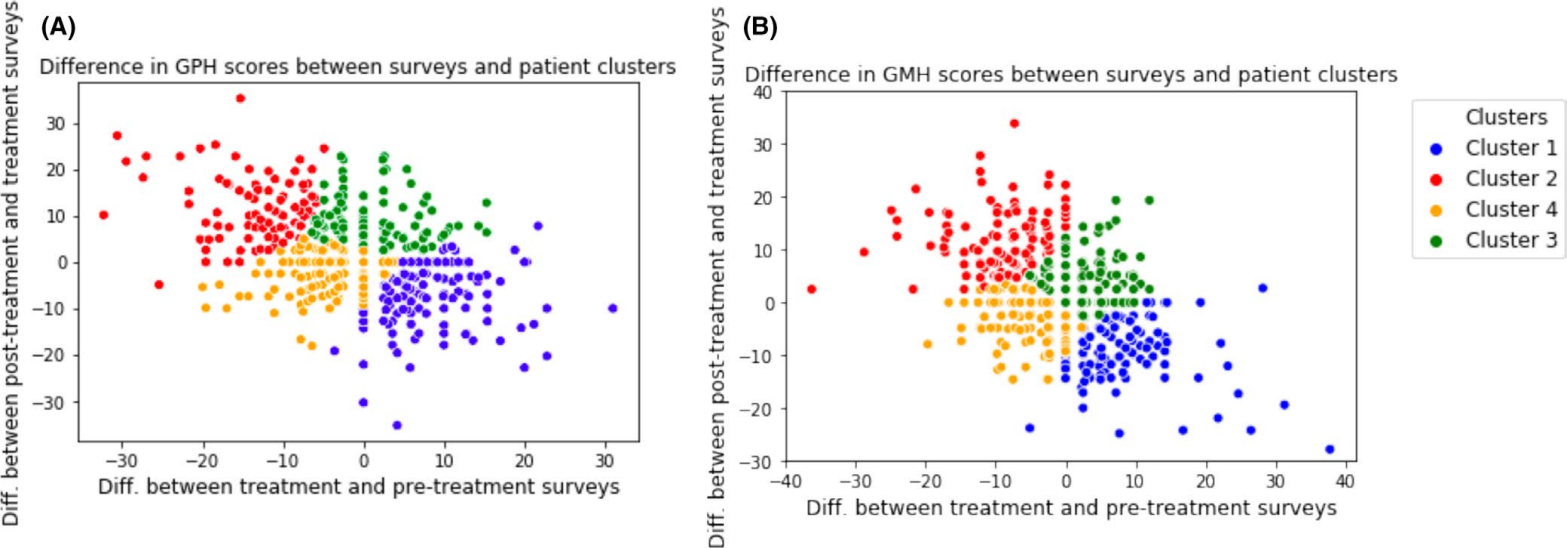
Distributions of difference between survey trajectories for all patients, with each point corresponding to a trajectory and color‐coding representing cluster membership for physical and mental health scores

**FIGURE 3:**
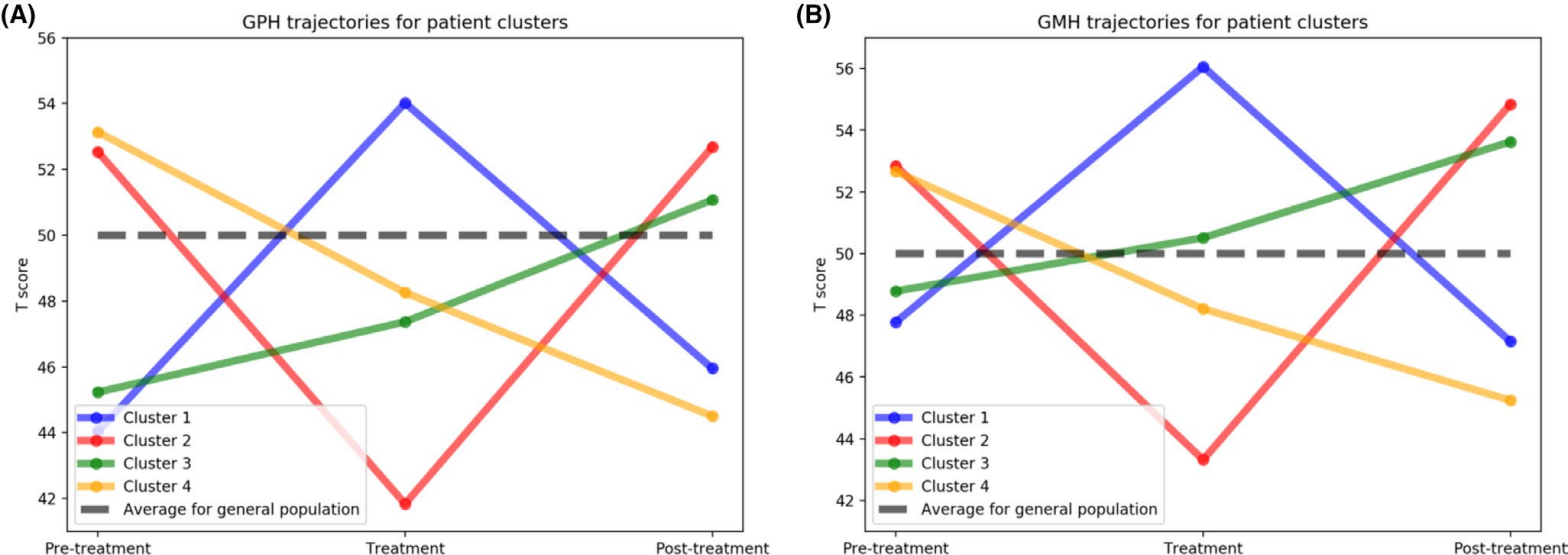
T‐score Trajectories across the pre‐treatment, treatment, and post‐treatment time periods for physical and mental health scores

To gain an understanding of patient‐reported outcomes over the course of therapy, we applied k‐means clustering to classify score trajectories before, during, and after chemotherapy. The K‐means clustering algorithms identified four unique patient trajectory clusters (Figure 2A,B). The plotted t‐scores from the four patient trajectory clusters are presented in Figure 3A,B. Cluster 1 (*n* = 135, ‘Temporary Improvers’) included patients who had increased GPH scores during treatment. Their T‐scores were below the population average in pre‐treatment (44.0, SD 8.7), scored above the population average during treatment (54.0, SD 7.8), and then deteriorated on their post‐treatment survey (46.0, SD 8.2). Cluster 2 (*n *= 147, ‘Temporary deteriorators’) appeared to be temporarily harmed by treatment. In this cluster, patients had above‐average scores pre‐treatment (52.5, SD 9.0), declined during treatment (41.8, SD 7.7), and had again above baseline scores post‐treatment (52.7, SD 8.7). Cluster 3 (*n* = 245, ‘Inexorable improvers’) began treatment with low baseline T‐scores (45.2, SD 8.3) that remained stable during treatment (47.4, SD 7.9) and then improved after treatment (51.1, SD 8.2). Cluster 4 (*n *= 214, ‘Inexorable deteriorators’) had above average *t*‐scores at pre‐treatment (53.1, SD 8.0) but steadily declined during (48.3, SD 8.2) and after treatment (44.5, SD 7.9). There was a similar pattern of scores for the GMH trajectories. Across physical score cluster types, there were significant differences in insurance status and age at diagnosis. For GPH, the inexorable deteriorators cluster had the highest proportion of patients with public insurance (57.5%) and tended to be older than other clusters (61.0 years, SD 13.0). There were no significant differences in clinical or demographic characteristics among mental health clusters. (Table S1).

## 4 DISCUSSION

Routine collection of PROs throughout a cancer patient’s chemotherapy journey provides a unique opportunity to examine patient experience during chemotherapy treatment in a real‐world setting. Using the PROMIS PRO tool linked to the EHR, we found that the majority of adults receiving outpatient chemotherapy did not show significant changes in their physical and mental health 12‐months post‐treatment compared to pre‐treatment scores. During chemotherapy, however, variation in patients’ physical and emotional well‐being was dramatic. We identified four unique novel trajectory clusters: Temporary improvers, Temporary deteriorators, Inexorable improvers, and Inexorable deteriorators. These novel trajectories could be leveraged to guide personalized supportive interventions to improve a patient’s chemotherapy experience. These findings have important implications for cancer care since PRO collection is now a requirement for CMS’ Oncology Care First model and lays the groundwork for future studies.

In this study, we found that, on average, cancer patients’ GPH and GMH scores are similar pre‐treatment and 12‐months post‐treatment and did not differ greatly from the general population average. These results are similar to single‐sample cross‐sectional studies comparing both breast cancer survivors and young adults with cancer to control population samples with no history of cancer.^21^, ^43^ These studies postulated patient resilience and better functioning as the primary reasons for PROMIS scores that do not differ from reference populations. The current study extends these findings to examine patients during and immediately after chemotherapy. We also identified patterns of change during treatment, which were often transient and resolved with the completion of chemotherapy. Though we report here on one of the largest collections of real‐world PRO data (as measured by PROMIS), this longitudinal cohort was too small to predict PROs accurately based on variables such as comorbidity number, socioeconomic status, stage, or use of potentially curative adjuvant treatment. Furthermore, it is also important to consider that the PROMIS global health measures may not be sensitive enough to assess key disease‐ or treatment‐specific symptoms, but that perhaps they are more generic measures for monitoring the quality of life in oncology patients.^12^, ^44^

Although patients had minimal changes between pre‐, intra‐, and post‐treatment scores, we were able to identify the treatment period as an important factor associated with a decline in well‐being during the course of chemotherapy. Declining physical health score was associated with Asian race, Hispanic ethnicity, Medicaid insurance, Medicare insurance, a diagnosis of anxiety/depression, palliative treatment, and stage III cancer compared to reference groups. We also found that a decline in mental health score was associated with Asian race, Hispanic ethnicity, Medicaid insurance, Medicare insurance, a diagnosis of anxiety/depression, palliative treatment, and lung cancer relative to their reference groups. Others have reported similar results, often finding an association of anxiety/depression, race/ethnicity, and advanced cancers with lower physical and mental health scores during cancer treatment.^22^, ^24^, ^33^, ^45^ The current results identify a population sample that may be particularly vulnerable to poor mental and physical health outcomes and this information can be used to guide resource allocation or identify these ‘higher risk’ groups prior to chemotherapy initiation for additional support. Furthermore, these data demonstrate the feasibility of collecting population‐based data to predict trends in oncology patients’ physical and psychological well‐being following chemotherapy treatment and also highlight the benefit of screening patients’ physical and mental health prior to treatment to establish a baseline and inform interventions. Such efforts should guide the testing of personalized supportive interventions to improve patients’ quality of life during and after chemotherapy.

While patients’ pre‐ and post‐treatment health scores were similar, patient experiences during chemotherapy were diverse between trajectory clusters. Many others have documented the adverse effect of chemotherapy on a patient’s physical and mental well‐being.^23^, ^45^, ^46^ However, using the standardized PROMIS survey across multiple time points, we found treatment trajectories clustered into four distinct patient profiles: Temporary improvers, Temporary deteriorators, Inexorable improvers, and Inexorable deteriorators. Temporary improvers reported higher physical and mental health scores during treatment than before and after treatment. Inexorable Improvers reported higher physical and mental health scores during treatment and after treatment compared to their pre‐treatment scores. Temporary Deteriorators had declines in PROMIS scores while on treatment, but then returned to baseline. Finally, Inexorable deteriorators had a linear decline of physical and mental health during and post‐treatment. Inexorable deteriorators tended to be older and publicly insured compared to the other clusters. Other distinguishing characteristics of clusters appeared only for physical health and included age at diagnosis and insurance status. Identification of distinct patient trajectory clusters during chemotherapy is novel. Ultimately, this broader characterization could help in therapy selection and in the identification of patients requiring supportive care during and after treatment.

We were not able to predict the four different patient trajectories accurately. This indicates that the variation we captured in physical and mental health scores during chemotherapy is likely not explained by standard patient demographic features such as age at diagnosis, race/ethnicity, and insurance. Instead, variable patient experiences during chemotherapy are likely due to other causes, such as differences in the metabolism of chemotherapeutic agents, genetic variations that influence the effects of chemotherapy on specific organ sites, family support during treatment, baseline personality traits,^47^ or even perspectives on healthcare.y^48^ While the current results demonstrate that it is important to understand not only a snapshot of a patient’s symptoms during cancer treatment, but rather their health trajectory and overall care experience, they also highlight the need for future research into the effects of chemotherapy treatment on patient‐centered outcomes.

While every effort was made to minimize bias, there are limitations to this study. Since these results were derived from a single geographic area, they may not be generalizable to the broader U.S. population. However, the inclusion of both academic and community healthcare settings increased the diversity of this study and generalizability of its results. The PROMIS surveys were collected via an internet‐based patient portal as well as on paper‐based forms, and therefore the current results might not apply to populations using only paper‐based surveys. However, these surveys were collected outside of a controlled trial and therefore offer insight into the effects of routine cancer care on GPH and GMH. Finally, as the data were collected retrospectively, loss to follow‐up resulted in a low number of patients with a survey at all three study timepoints. Yet, to our knowledge, this is the largest longitudinal study to date of PROs in cancer patients using PROMIS surveys. When we compared our cohort with patients who did not complete PROMIS surveys, we found important differences that highlight the need for continued outreach interventions. This analysis of patient’s reported trajectories during real‐world chemotherapy administration has strong potential to inform improvements to patient‐centered cancer care.

Precision oncology care requires the integration and analysis of PROs. Now, as CMS has moved toward the Oncology Care First model and requires PRO collection, we have the opportunity to leverage these data to improve symptom detection and control and quality of life, which might ultimately improve high‐value care and survival. Understanding the physical and mental health needs of a patient and using tailored interventions to improve predicted outcomes is the future of healthcare delivery. Using routinely collected PROMIS surveys in a real‐world setting, we are able to determine important predictors of declines in physical and mental well‐being. We further defined four novel patient trajectories during chemotherapy yet were not able to accurately predict these trajectories. These findings have implications for future studies that may identify novel factors associated with the patient experience and individual patients who may need support. Such evidence is essential to identify and intervene upon patients most vulnerable to adverse experiences with chemotherapy.

## CONFLICT OF INTEREST

The authors declare that they have no known competing financial interests or personal relationships that could have appeared to influence the work reported in this paper.

## Supporting information

Supplementary Material
