# Supplementary material for: Diverse patient trajectories during cytotoxic chemotherapy: Capturing longitudinal patient‐reported outcomes
Source: Cancer Med. 2021 Jul 13;10(17):5783–93. doi: 10.1002/cam4.4124 (PMC8419778; doi:10.1002/cam4.4124)

**Supplementary Table 1:** Codes used to define chemotherapy and depression

**Supplementary Table 2:** Characteristics of PROMIS Score Trajectory Clusters

**Supplementary Figure 1:** CONSORT diagram for cohort selection

**Supplementary Figures 2A and 2B:** A, Physical health score trajectories by cancer type. B, Mental health score trajectories by cancer type

**Supplementary Table 1:** Codes used to define chemotherapy and depression

|  | Chemotherapy | Depression |
| --- | --- | --- |
| ICD-9 | 99.25 | 296.2, 296.22, 296.23, 296.24, 296.25, 296.26, 296.20, 296.3, 296.32, 296.33, 296.34, 296.35, 296.36, 296.30, 311 |
| ICD-10 | Z51.11 | F32, F32.0, F32.1, F32.2, F32.3, F32.8, F32.9, F41.2, F33, F33.0, F33.1, F33.2, F33.4, F33.8, F33.9 |
| CPT | 96401, 96402, 96405, 96406, 96409, 96411, 96413, 96415, 96416, 96417, 96420, 96422, 96423, 96425, 96440, 96445, 96446, 96450, 96542, 96549 |  |

**Supplementary Table 2:** Characteristics of PROMIS Score Trajectory Clusters

|  | GPH | | | | GMH | | | |
| --- | --- | --- | --- | --- | --- | --- | --- | --- |
|  | Temporary Improvers (n=135) | Temporary Deteriorators (n=147) | Inexorable Improvers (n=245) | Inexorable Deteriorators (n=214) | Temporary Improvers (n=126) | Temporary Deteriorators (n=285) | Inexorable Improvers (n=143) | Inexorable Deteriorators (n=187) |
| **Sex** |  |  |  |  |  |  |  |  |
| *Female* | 89 (65.9) | 87 (59.2) | 140 (57.1) | 122 (57.0) | 75 (59.5) | 167 (58.6) | 85 (59.4) | 111 (59.4) |
| *Male* | 46 (34.1) | 60 (40.8) | 105 (42.9) | 92 (43.0) | 51 (40.5) | 118 (41.4) | 58 (40.6) | 76 (40.6) |
| **Age*** | 59.8 (12.8) | 58.8 (12.7) | 57.3 (13.4) | 61.0 (13.0) | 59.1 (13.6) | 59.1 (13.0) | 58.2 (12.4) | 59.9 (13.5) |
| **Race/Ethnicity** | |  |  |  |  |  |  |  |
| *Non-Hispanic white* | 79 (58.5) | 99 (67.3) | 160 (65.3) | 139 (65.0) | 75 (59.5) | 190 (66.7) | 93 (65.0) | 119 (63.6) |
| *Asian* | 39 (28.9) | 25 (17.0) | 41 (16.7) | 36 (16.8) | 27 (21.4) | 48 (16.8) | 30 (21.0) | 36 (19.3) |
| *Other* | 17 (12.6) | 21 (14.3) | 44 (18.0) | 34 (15.9) | 23 (18.3) | 45 (15.8) | 18 (12.6) | 30 (16.0) |
| **Insurance*** | |  |  |  |  |  |  |  |
| *Private* | 63 (46.7) | 85 (57.8) | 126 (51.4) | 90 (42.1) | 58 (46.0) | 129 (45.3) | 82 (57.3) | 95 (50.8) |
| *Public* | 71 (52.6) | 59 (40.1) | 119 (48.6) | 122 (57.0) | 67 (53.2) | 155 (54.4) | 59 (41.3) | 90 (48.1) |
| **Depression diagnosis** | |  |  |  |  |  |  |  |
| *Diagnosis* | 28 (20.7) | 29 (19.7) | 48 (19.6) | 39 (18.2) | 26 (20.6) | 58 (20.4) | 29 (20.3) | 31 (16.6) |
| *No Diagnosis* | 107 (79.3) | 118 (80.3) | 197 (80.4) | 175 (81.8) | 100 (79.4) | 227 (79.6) | 114 (79.7) | 156 (83.4) |
| **Stage** |  |  |  |  |  |  |  |  |
| *I* | 31 (23.0) | 39 (26.5) | 58 (23.7) | 53 (24.8) | 24 (19.0) | 74 (26.0) | 38 (26.6) | 45 (24.1) |
| *II* | 31 (23.0) | 32 (21.8) | 53 (21.6) | 52 (24.3) | 29 (23.0) | 62 (21.8) | 34 (23.8) | 43 (23.0) |
| *III-IV* | 46 (34.1) | 52 (35.4) | 81 (33.1) | 66 (30.8) | 48 (38.1) | 85 (29.8) | 48 (33.6) | 64 (34.2) |
| **Treatment goal** | |  |  |  |  |  |  |  |
| *Curative* | 90 (66.7) | 114 (77.6) | 184 (75.1) | 155 (72.4) | 90 (71.4) | 219 (76.8) | 106 (74.1) | 128 (68.4) |
| *Palliative* | 45 (33.3) | 33 (22.4) | 61 (24.9) | 59 (27.6) | 36 (28.6) | 66 (23.2) | 37 (25.9) | 59 (31.6) |
| **Cancer Type** | |  |  |  |  |  |  |  |
| *Breast* | 43 (31.9) | 52 (35.4) | 52 (21.2) | 68 (31.8) | 37 (29.4) | 70 (24.6) | 50 (35.0) | 58 (31.0) |
| *Lymphoma/Leukemia* | 25 (18.5) | 17 (11.6) | 52 (21.2) | 40 (18.7) | 25 (19.8) | 65 (22.8) | 17 (11.9) | 27 (14.4) |
| *Genitourinary* | 10 (7.4) | 12 (8.2) | 21 (8.6) | 20 (9.3) | 15 (11.9) | 23 (8.1) | 11 (7.7) | 14 (7.5) |
| *Other* | 57 (42.2) | 66 (44.9) | 120 (49.0) | 86 (40.2) | 49 (38.9) | 127 (44.6) | 65 (45.5) | 88 (47.1) |

**Supplementary Table 3:** Sensitivity analysis comparing study cohort to chemotherapy patients without PROMIS surveys

| **Characteristic** | **non-PROMIS patients** | **PROMIS patients** | p-value |
| --- | --- | --- | --- |
| **Age at diagnosis, mean (SD)** | 57.7 (16.3) | 59.1 (13.1) | 0.024 |
| **Sex, N(%)** |  |  | <0.001 |
| *Male* | 3141 (49.4) | 303 (40.9) |  |
| *Female* | 3223 (50.6) | 438 (59.1) |  |
| **Race/Ethnicity, N(%)** |  |  | <0.001 |
| *Non-Hispanic white* | 3399 (53.4) | 477 (64.4) |  |
| *Hispanic* | 826 (13.0) | 61 (8.2) |  |
| *Asian* | 1325 (20.8) | 141 (19.0) |  |
| *Other* | 711 (11.2) | 55 (7.4) |  |
| **Insurance, N(%)** |  |  | <0.001 |
| *Private* | 3017 (47.4) | 364 (49.1) |  |
| *Medicare* | 2685 (42.2) | 351 (47.4) |  |
| *Medicaid* | 662 (10.4) | 20 (2.7) |  |
| **Depression diagnosis, N(%)** |  |  | 0.912 |
| *Diagnosis* | 1226 (19.3) | 144 (19.4) |  |
| *No Diagnosis* | 5138 (80.7) | 597 (80.6) |  |
| **Stage at diagnosis, N(%)** |  |  | <0.001 |
| *I* | 895 (14.1) | 181 (24.4) |  |
| *II* | 1098 (17.3) | 168 (22.7) |  |
| *III* | 785 (12.3) | 117 (15.8) |  |
| *IV* | 1721 (27.0) | 128 (17.3) |  |
| **Cancer Type, N(%)** |  |  | <0.001 |
| *Breast* | 1006 (15.8) | 215 (29.0) |  |
| *Lymphoma/Leukemia* | 1638 (25.7) | 134 (18.1) |  |
| *Gastrointestinal* | 662 (10.4) | 76 (10.3) |  |
| *Head and Neck* | 484 (7.6) | 75 (10.1) |  |
| *Genitourinary* | 702 (11.0) | 63 (8.5) |  |
| *Lung* | 617 (9.7) | 54 (7.3) |  |
| *Gynecologic* | 398 (6.3) | 41 (5.5) |  |
| *Skin* | 184 (2.9) | 31 (4.2) |  |
| *Hepatobiliary/Pancreatic* | 387 (6.1) | 30 (4.0) |  |
| *Other* | 227 (3.6) | 22 (2.9) |  |

**Supplementary Figure 1.** CONSORT diagram for cohort selection


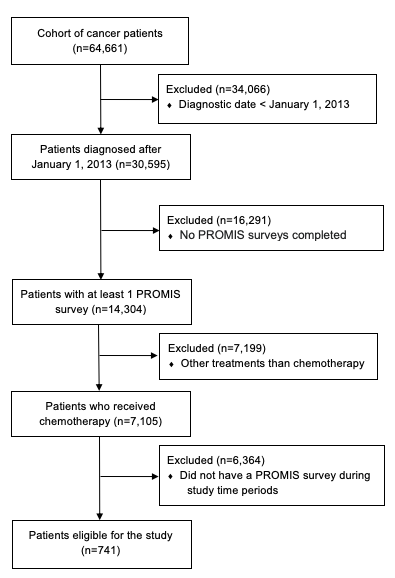

Supplement: Supplementary file 1 — Supplementary Material [file CAM4-10-5783-s001.docx]
